# Surface-Modified Highly Biocompatible Bacterial-poly(3-hydroxybutyrate-*co*-4-hydroxybutyrate): A Review on the Promising Next-Generation Biomaterial

**DOI:** 10.3390/polym13010051

**Published:** 2020-12-25

**Authors:** Jun Meng Chai, Tan Suet May Amelia, Govindan Kothandaraman Mouriya, Kesaven Bhubalan, Al-Ashraf Abdullah Amirul, Sevakumaran Vigneswari, Seeram Ramakrishna

**Affiliations:** 1 Faculty of Science and Marine Environment, Universiti Malaysia Terengganu, Kuala Nerus 21030, Terengganu, Malaysia; junmeng1994@gmail.com (J.M.C.); p3838@pps.umt.edu.my (T.S.M.A.); mouriyagk0172@gmail.com (G.K.M.); kesaven@umt.edu.my (K.B.); 2 School of Biological Sciences, Universiti Sains Malaysia, Penang 11800, Malaysia; 3 Center for Nanofibers and Nanotechnology, Department of Mechanical Engineering, National University of Singapore, Singapore 117581, Singapore

**Keywords:** surface modifications, polyhydroxyalkanoates, poly(3-hydroxybutyrate-*co*-4-hydroxybutyrate), biomedical applications, tissue engineering, biocompatible

## Abstract

Polyhydroxyalkanoates (PHAs) are bacteria derived bio-based polymers that are synthesised under limited conditions of nutritional elements with excess carbon sources. Among the members of PHAs, poly(3-hydroxybutyrate-*co*-4-hydroxybutyrate) [(P(3HB-*co*-4HB)] emerges as an attractive biomaterial to be applied in medical applications owing to its desirable mechanical and physical properties, non-genotoxicity and biocompatibility eliciting appropriate host tissue responses. The tailorable physical and chemical properties and easy surface functionalisation of P(3HB-*co*-4HB) increase its practicality to be developed as functional medical substitutes. However, its applicability is sometimes limited due to its hydrophobic nature due to fewer bio-recognition sites. In this review, we demonstrate how surface modifications of PHAs, mainly P(3HB-*co*-4HB), will overcome these limitations and facilitate their use in diverse medical applications. The integration of nanotechnology has drastically enhanced the functionality of P(3HB-*co*-4HB) biomaterials for application in complex biological environments of the human body. The design of versatile P(3HB-*co*-4HB) materials with surface modifications promise a non-cytotoxic and biocompatible material without inducing severe inflammatory responses for enhanced effective alternatives in healthcare biotechnology. The enticing work carried out with P(3HB-*co*-4HB) promises to be one of the next-generation materials in biomedicines which will facilitate translation into the clinic in the future.

## 1. Introduction

Amongst the many different biocompatible bioplastics studied, polyhydroxyalkanoates (PHAs) are arguably the closest to common plastics in terms of mechanical strength and processability. The mechanical properties of PHAs is to date is the most superior to be developed as biomaterials and are widely used in medical applications, especially tissue engineering [1,2,3]. Besides their biocompatibility, PHAs have attracted considerable interest due to their biodegradability, sustainability and thermo processability [4,5,6], and the degradation of PHAs do not cause tissue response in living organisms [7]. PHAs are generally produced by microorganisms as intracellular carbon and energy sources under excess carbon [8]. Nevertheless, the main challenge in large-scale PHA production and commercialisation is the cost of production which is often related to carbon feedstock, production yield and extraction processes [9,10]. On-going research focuses on PHA production from wastes and inexpensive substrates from agriculture and food industries [11,12] as well as recovery and purification using various chemical and physical processes [13]. 

The common types of PHA which exhibit good biocompatibility are composed of 3-hydroxybutyrate, 4-hydroxybutyrate, 3-hydroxyvalerate or 3-hydroxyhexanaote monomers [14]. However, poly(3-hydroxybutyrate-*co*-4-hydroxybutyrate) [P(3HB-*co*-4HB)] is the most widely studied and has gained attention as a potential biomaterial in biomedical research areas. Importantly, the degradation of the copolymer resulted in the synthesis of 4-hydroxybutyrate (4HB) monomer which is a normal constituent of the mammalian body [15]. In addition, the presence of 4HB monomer in the copolymer provides flexible properties for suitable use as implantable medical materials [16]. Interestingly, the surface properties of P(3HB-*co*-4HB) can be further enhanced via the surface modification approach. 

**Figure 1:**
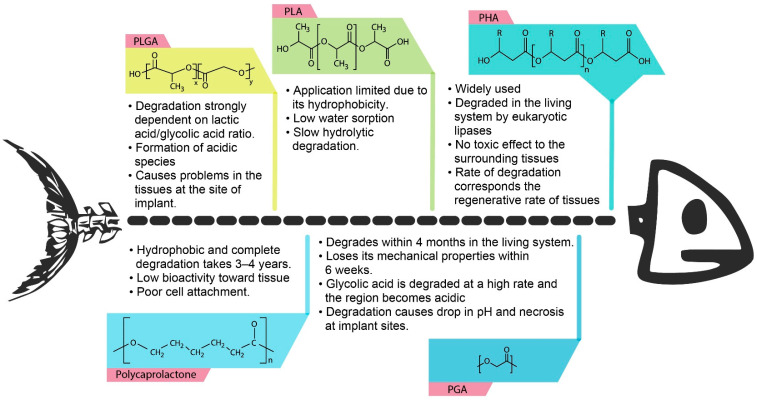
The various types of biodegradable polymers widely used as biomaterials are distinguished based on their behaviour on implantation sites and biodegradation of these biopolymers during implantation.

Besides PHA, some other synthetic biopolymers that have been widely used as biomaterials include polyglycolic acid (PGA), poly(L-lactic acid) (PLLA) and poly(DL-lactic-*co*-glycolic acid) (PLGA) and polyurethane [17,18]. However, the long-term biostability of these synthetic biopolymers has proved an obstacle in medical applications. Moreover, these polymers degrade into lactic acid and glycolic acid which accumulate in the body, and as they are mostly acidic, they reduce the local pH, impacting the delivery and absorption of proteins and other bioactive molecules, thus inducing severe inflammatory responses, even causing cell and tissues necrosis at the implant sites [19,20,21]. Figure 1 shows the different types of biodegradable polymers commonly used as biomaterials distinguished according to their behaviour on implantation.

Tissue engineering has been considered the ideal strategy for regenerative medicine in cardiology. It is an interdisciplinary field combining biomedical, biotechnological and engineering techniques that aim to maintain, regenerate or replace tissues or organs. Advances in tissue engineering are evident, and the application of this technology to the regeneration of tissues has been increasingly explored and has presented encouraging results. In this regard, this review aims to intricately discuss the multiple approaches taken in improving the surface functionality of P(3HB-*co*-4HB) and elaborate its targeted applications in various biomedical aspects to date. 

## 2. Polyhydroxyalkanoates (PHAs) 

**Figure 2:**
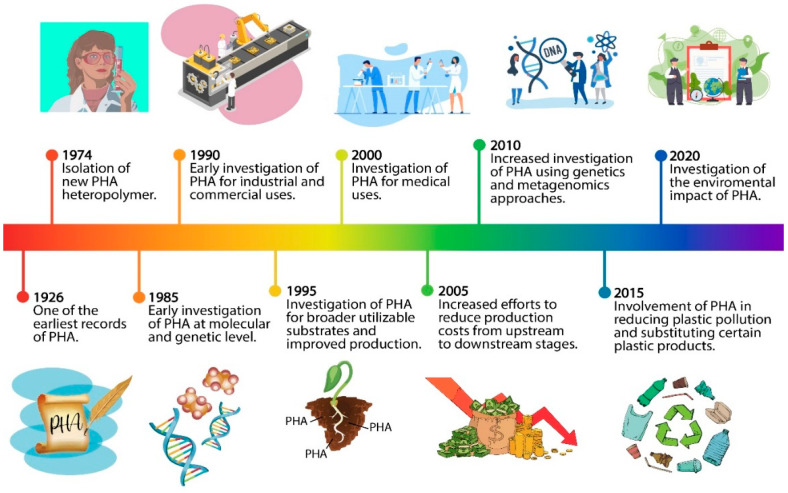
The timeline of polyhydroxyalkanoate (PHA) that indicates the past and current major development trends in science and applications [34,35,36,37,38,39].

PHAs can be synthesised by various microorganisms as carbon and energy sources intracellularly under stress conditions [22,23]. They are one of the most promising biopolymers due to their excellent biocompatibility, biodegradability and non-toxic degradation [24,25,26]. Indeed, PHAs are the only bio-based polymers that are entirely produced and degraded by living cells. They can be degraded into carbon dioxide and water by various microorganisms using their own secreted PHA depolymerases and eukaryotic enzymes such as lipase enzymes [4]. The historical timeline for PHA development is shown in Figure 2.

**Figure 3:**
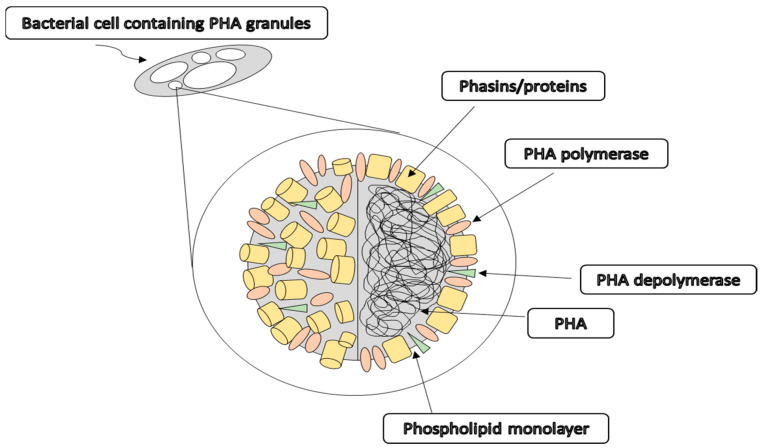
The schematic overview of a PHA granule accumulated intracellularly in the bacterial cells as carbon and energy sources under stress conditions [27].

PHAs were first discovered in *Bacillus megaterium* by the French scientist, Maurice Lemoigne, in 1926, who observed the production of an intracellular polymer of hydroxybutyrate monomers, later named polyhydroxybutyrate [P(3HB)], one of the most common PHAs synthesised by bacteria [27,28]. In 1968, Griebel investigated the composition of P(3HB) granules of *B. megaterium* for the first time, concluding that they consisted of 97.7% polymer, 1.87% protein, and 0.46% lipids or phospholipids [29]. Thus, PHA polymers occupy more than 90% of the bacterial cell dry weight as shown in Figure 3. Moreover, the PHA granules are localised in the cytoplasm, existing as discrete inclusions of approximately 0.2 ± 0.5 mm in diameter [30]. The core consists of PHA polymers surrounded by a phospholipid monolayer and a few integrated proteins, such as PHA depolymerase, PHA polymerase and phasins or proteins [27].

**Figure 4:**
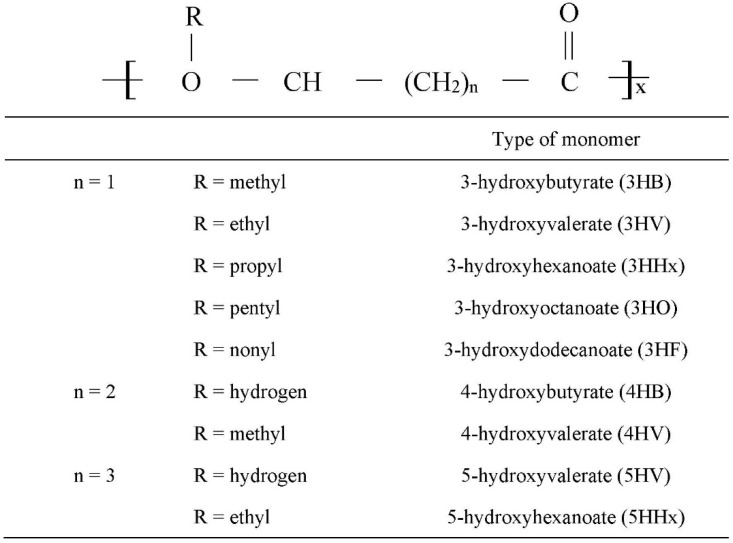
General chemical structure of PHA where the R configuration is located at the hydroxyl-substituted carbon atom whereas n refers to the size of the alkyl group [33].

PHAs are polymers of carbon, oxygen and hydrogen mainly arranged in a linear structure and consist of head-to-tail polymers composed of 3-hydroxy fatty acid monomers [31,32]. Figure 4 shows the general chemical structure of PHA monomers, where the R configuration is located at the hydroxyl-substituted carbon atom and n refers to the size of the alkyl group [24,33]. The polymers are constructed by the formation of ester bonds via the condensation of the carboxyl group of one monomer with the hydroxyl group of the adjacent monomer. There are various types of PHA, such as poly[3-hydroxybutyrate] [P(3HB)], poly(4-hydroxybutyrate) [P(4HB)], poly[3-hydroxybutyrate-*co*-4-hydroxybutyrate] [P(3HB-*co*-4HB)], poly[3-hydroxybutyrate-*co*-3-hydroxyvalerate] [P(3HB-*co*-3HV)] and poly[(3-hydroxyoctanoate-*co*-3-hydroxyhexanoate] [P(3HO-*co*-3HHx)].

### 2.1. Properties of Polyhydroxyalkanoates (PHAs)

PHAs have garnered attention from researchers worldwide because of their desirable properties, such as biodegradable, non-toxic, biocompatible, thermoplastic, eco-friendly, sustainable, adjustable chemical and physical properties [4,24,40], making them excellent candidates to replace petrochemical plastics. The wide range of properties is presented in Table 1. The production of PHAs does not cause any harmful effects, hence is considered environmentally friendly to both organisms and the environment [7]. Furthermore, PHAs can be synthesised on a large-scale for sustainable development without causing any deleterious environmental impact.

One of the important properties of PHAs that has become the focus of polymer research is their biodegradability [7,25]. PHAs are degraded completely into CO_2_ and H_2_O via natural microbiological mineralisation [14]. It has been reported that PHA depolymerases, PHA hydrolases and lipases are capable of degrading PHAs through hydrolytic and enzymatic degradation, thereby reducing the problem of plastic waste accumulation [4,23,45]. Moreover, Abe and Doi [46] concluded that the rate of degradation is also affected by the characteristics of the biopolymer, including their structure, chemical composition and crystallinity.

PHAs also possess a broad range of physical and mechanical properties from hard crystalline to elastomeric properties. The physical properties of PHAs depend on the different monomers introduced into the polymer chains which can be varied to suit the specific needs for various applications. Thus, PHAs can be tailored to specific needs by regulating the type and molar composition of the monomer introduced. In addition, PHA polymers composed solely of SCL monomer units usually have thermoplastic properties, whereas polymers composed of MCL subunits normally have elastomeric properties [47]. The mixed chain length PHAs possess a range of physical properties depending on the different monomer composition, with polymers with a low percentage of monomers being more elastomeric [8].

### 2.2. P(3HB-co-4HB) Producing Bacteria

PHA producers include a wide range of bacteria and archaea which reside in diverse ecological niches [48]. These bacteria and archaea accumulated PHA in intracellular granules to serve as storage reserve materials [49]. More than 300 different bacteria have been identified as microbial PHAs producers, most of which are Gram-negative bacteria [33]. However, there are also certain gram-positive bacteria, such as *Bacillus megaterium*, *Corynebacterium glutamicum* and *Microlunatus phosphovorus*, that possess the ability to produce PHAs intracellularly [50].

Generally, most bacteria synthesise PHAs with limited essential nutrients but excess carbon sources. In conditions of limited nutrients, the bacteria accumulate PHA granules in response to starvation as an energy store for survival [51]. However, there are certain bacteria, such as recombinant *Escherichia coli* and *Alcaligenes latus*, that do not require nutrient deficiency for the accumulation of PHAs. The bacteria which can synthesise different classes of PHAs are shown in Table 2.

## 3. Synthesis of Poly(3-hydroxybutyrate*-co-*4-hydroxybutyrate) [P(3HB*-co-*4HB)]

**Figure 5:**
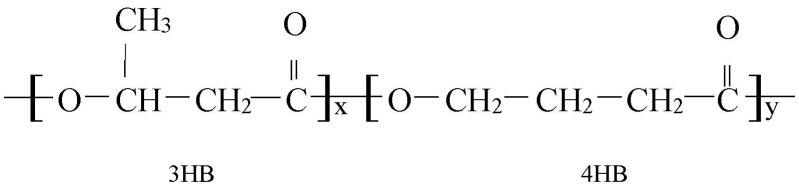
Chemical structure of P(3HB*-co-*4HB) where x and y refer to the number of repeating units of 3HB and 4HB monomer composition respectively [58].

The synthesis of PHA monomers by bacterial strain is often dependent on the structure of carbon substrate. Co-monomers are incorporated by the addition of precursors, however, in certain circumstances the precursor can also act as the sole carbon substrate for the synthesis of more than one monomer. Among the variety of PHAs, P(3HB*-co-*4HB) is noted as the most biocompatible material that has gained the most attention among researchers [58]. P(3HB*-co-*4HB) consists of two different monomers including 3HB and 4HB monomers as shown in Figure 5. The physical and mechanical properties of the copolymers are influenced by the composition of the monomer, which can be manipulated by varying the monomer composition. Therefore, the incorporation of 4HB monomer units into the 3HB chain has greatly improved the thermal, crystalline and mechanical properties of PHA. In addition, the in vivo degradation rate of P(3HB*-co-*4HB) is relatively high compared with other PHAs and can be controlled by the 4HB fraction. P(3HB*-co-*4HB) is especially attractive as it is capable of eukaryotic lipase hydrolyzation into natural metabolites in humans [59]. Also, P(3HB*-co-*4HB) has desirable mechanical properties for applications in the medical and pharmaceutical fields.

**Figure 6:**
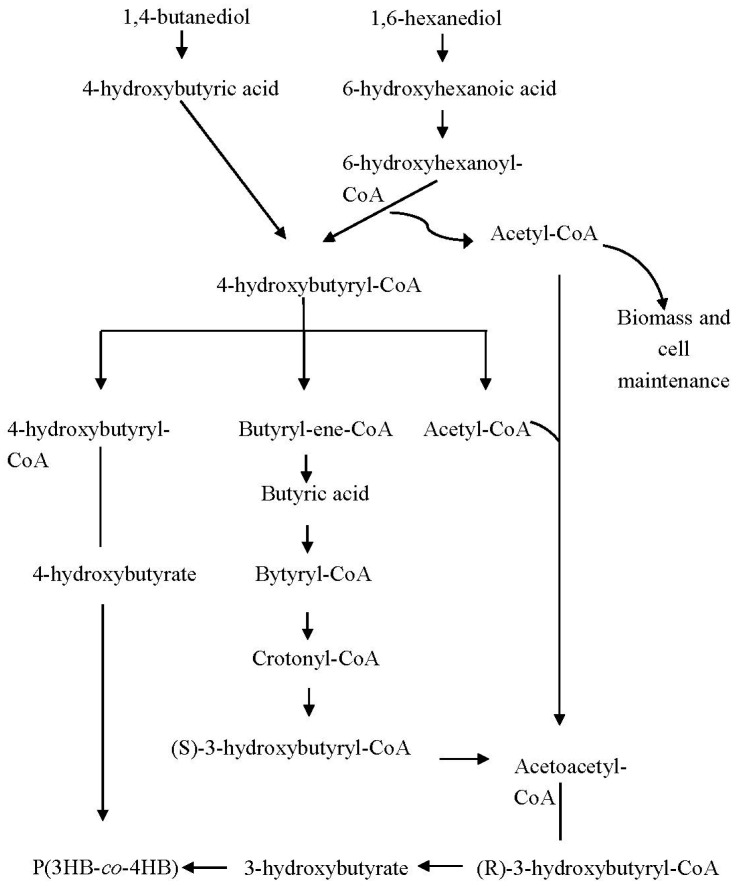
The biosynthesis pathway for the intracellular production of P(3HB-*co*-4HB) [64].

In the early stage of the polymer research, Doi et al. [60] synthesised P(3HB*-co-*4HB) copolymer since 4-hydroxybutyric acid was used as the sole carbon substrate. Subsequently, they found that the synthesis of this copolymer can also be achieved with different carbon sources such as γ-butyrolactone, 1,4-butanediol and 4-chlorobutyric acid [61]. Nowadays, the study of P(3HB*-co-*4HB) production has been improved by the combination of various carbon sources to increase the 4HB monomer. Incorporation of the 4HB monomer is achieved by adding precursor substrates such as 4-hydroxybutyric acid, γ-butyrolactone and ω-alkanediols [62,63]. Figure 6 shows the biosynthesis pathway of P(3HB*-co-*4HB) copolymer utilising both 1,4-butanediol and 1,6-hexanediol as carbon precursors, which are composed of four and six carbon atoms, respectively.

In the biosynthesis pathway, 1,4-butanediol is first oxidised to 4-hydroxybutyric acid, subsequently into 4-hydroxybutyryl-CoA [54]. In concept, ω-alkanediols are oxidised into the corresponding ω-hydroxy fatty acid, then converted into coenzyme A thioester [65]. In this regard, 1,6-hexanediol is oxidised into 6-hydroxyhexanoic acid, then converted into 6-hydroxyhexanoyl-CoA, which is converted into 4-hydroxybutyryl-CoA via β-oxidation. In this stage, 6-hydroxyhexanoyl-CoA which possesses six carbon atoms is required to be reduced to form 4-hydroxybutyryl-CoA with four carbon atoms before polymerising by PHA synthase. Thus, β-oxidation eliminates two carbon atoms, simultaneously releasing acetyl-CoA, a vital intermediate for bacterial growth, biomass and cell maintenance as well as 3HB monomer formation [64]. 4-Hydroxybutyryl-CoA is directly polymerised by PHA synthase since it is not a chiral intermediate compared to 3-hydroxybutyryl-CoA [65]. A part of 4-hydroxybutyryl-CoA is converted into 4HB, while another part undergoes a complex pathway converting it into 3-hydroxybutyryl-CoA, subsequently forming 3HB monomer, which incorporates with 4HB to produce P(3HB*-co-*4HB) [54].

### 3.1. Physical and Mechanical Properties of P(3HB-co-4HB)

The physical and mechanical properties of copolymer P(3HB*-co-*4HB) are affected by the monomer composition. Vigneswari et al. [66] determined that the glass transition temperature (Tg) of P(3HB*-co-*4HB) ranged from −22 to 1 °C, with a melting temperature (Tm) between 46 to 167 °C for a 4HB molar fraction from 16 mol% to 91 mol%. The average molecular weight (Mn) ranged from 68 × 10^3^ to 250 × 10^3^ Da, while the polydispersity index was between 1.3 to 2.1 [66]. It was reported that the crystallinity of P(3HB*-co-*4HB) ranged from 35% to 51% with the 4HB molar fraction from 6.1 to 48.3 mol% [67]. Meanwhile, the Young’s modulus of P(3HB*-co-*4HB) ranged between 4.12 to 817.50 MPa, the tensile strength between 4.92 to 15.74 MPa, and the elongation to break ranged from 13.10% to 311.47% with the range of 6.1 to 48.3 mol% 4HB monomer [67].

### 3.2. Biodegradability of P(3HB-co-4HB)

The biodegradability of P(3HB*-co-*4HB) is a great advantage over those petroleum-based plastics and can be controlled. Vigneswari and Amirul [68] demonstrated that P(3HB*-co-*4HB) nanofibre scaffold completely degraded in around four weeks, whereas it took three to four years to completely degrade synthetic polymers such as poly-ε-caprolactone (PCL) [69].

Since P(3HB*-co-*4HB) is often used as a biomaterial implanted inside the body, the mechanism of degradation in the living system is very important. The degradation rate of the biomaterial P(3HB*-co-*4HB) should be similar to the regenerative rate of the living tissues during implantation for successful implantation of the P(3HB*-co-*4HB) scaffold [14,70]. The biodegradability of P(3HB*-co-*4HB) is controllable so that it can be used effectively as a scaffold in different tissues as the regeneration rate of various tissues differs. Specifically, P(3HB*-co-*4HB) is degradable in vivo due to hydrolytic or lipase enzyme activities in the body, eventually breaking down into 3HB and 4HB monomers. The 3HB associates with the ketone body metabolism and is eliminated from the body [71], whereas 4HB is eliminated from the body primarily by the TCA cycle (tricarboxylic acid cycle) TGA cycle, eventually converted to carbon dioxide and water [15].

### 3.3. Biocompatibility of P(3HB-co-4HB)

Biocompatibility is defined as the capability of a biomaterial to adapt to the appropriate host responses in a particular application [14,31]. Hence, P(3HB*-co-*4HB) as a biomaterial should be biocompatible, that is, unable to elicit severe immune responses when introduced or implanted into the living tissues of the host organisms [7]. The elicitation of immune responses can slow down the tissue regeneration and healing process, even trigger rejection in the body [72]. Moreover, P(3HB*-co-*4HB) does not provoke immune reactions during degradation and is considered biocompatible [71]. Biocompatible P(3HB*-co-*4HB) serves as a substrate to form cell–scaffold interactions to promote cellular activities. According to a study by Mu et al. [73], bone marrow mesenchymal stem cells (BMSCs) seeded on the P(3HB*-co-*4HB) material showed good cell viability and growth, reflecting the good biocompatibility of (3HB-*co*-4HB) materials.

## 4. Surface Functionalisation of P(3HB*-co-*4HB)

The biomaterial surface plays a key role in inducing cell–biomaterial interactions within the biological system. 

**Figure 7:**
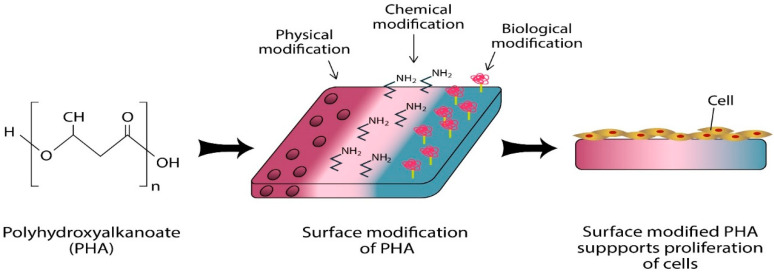
Schematic diagram of the various surface modifications of PHA to improve the surface properties of the biopolymer. Physical modification alters the morphology and physical properties of the surface; chemical modification creates functionalised surface by introducing functional groups; biological modification creates biofunctionalised surface by introducing biological molecules.

Nowadays, the surface properties of biomaterial can be improved via various modification methods which includes physical, chemical and biological methods as summarised in Figure 7. These modification techniques have been developed to alter the wettability, morphology and chemical properties of the surfaces of biomaterial to satisfy the requirements in the design of a functional scaffold [74]. The surface modification approaches include plasma modification, physical modification, chemical modification and biological modification.

Plasma modification is an effective surface treatment that selectively alters the physical and chemical properties of the biomaterial surface without influencing the original bulk properties of the biomaterial. It modifies the surface chemistry and topography of the surface. 

The P(3HB-*co*-4HB) polymer with Ar plasma pretreatment followed by poly(acrylic acid) grafting reaction showed an increased surface polarity, adhesion, proliferation and improved cells-material interactions [74,75]. Plasma modification using argon (Ar), hydrogen peroxide (H_2_O_2_) and water (H_2_O) may cause changes in the surface hydrophilicity or biodegradability, whereas the introduction of diverse functional groups by plasma treatment depends on the type of gas used, such as oxygen (O_2_), carbon dioxide (CO_2_), and ammonia (NH_3_) [76]. 

Physical modification techniques include facile blending, freeze-drying, porogen leaching, electrospinning and ultraviolet (UV) irradiation. Facile blending is a simple approach to fabricate new polymeric materials with enhanced properties. Vigneswari et al. [77] combined P(3HB*-co-*4HB) with collagen via facile blending to develop a P(3HB*-co-*4HB)-collagen blend scaffold, which exhibited enhanced adhesion of murine fibroblast cells (L929). The blending method can produce a porous scaffold which enhances cell proliferation.

Freeze drying is a physical modification using lyophilisation to fabricate a porous scaffold. It involves the dissolution of polymer materials in a suitable solvent, which are then cooled under the freezing point to evaporate the solidified solvent by sublimation to form the porous dry scaffold [78]. The merit of this method is that the pore size can be easily controlled by adjusting the freezing regime. Vigneswari et al. [43] synthesised an antimicrobial scaffold of the silver sulfadiazine blend/P(3HB-*co*-4HB)-collagen peptide biocomposite containing via freeze-drying, which enhanced the proliferation of osteosarcoma L929 cells, hence is a potential biomaterial.

Porogen leaching is another physical modification technique which creates grooves and microstructures on the biomaterial surface. This method involves casting a polymer solution with the porogen, a particle with specified size and shape used to fabricate a porous scaffold for tissue engineering. The porogen, such as salt particles or sodium bicarbonate (NaHCO_3_), is leached out with water to form the pores on the polymer surfaces [79]. The hydrophilicity and water uptake of degraded salt-leached film of P(3HB-*co*-4HB) were enhanced by salt-leached technique which enhanced the cell attachment and suitable for biomedical use as a scaffold [43,80].

Chemical modification is a promising approach to introduce desired functional groups via chemical etching onto the surface of the biomaterial. Generally, the chemical modification involves electrophilic or nucleophilic reactions at the introduced functional groups on the surface. The surface treatments by chemical methods break the ester bonds on the biomaterial surfaces to generate functional groups, thereby altering their surface chemical composition [74]. The chemical modification methods include base hydrolysis and aminolysis which introduce carboxyl groups and amine groups respectively onto the surface of the biomaterial [81]. These chemical modifications improve the hydrophilicity of scaffold surfaces, which in turn promotes cell attachment and proliferation are promising new classes of biocompatible PHA hybrid biomaterials which are of great interest for medical and therapeutic applications [82].

Furthermore, biological modification involves the attachment of biologically functional molecules on the biomaterial surfaces that governs the cellular responses. Biomacromolecules such as polysaccharides, proteins, proteoglycans and glycoproteins play a vital role as the biological cues for adherent cells. The integrins present on the cell membrane surface comprise extracellular matrix (ECM) proteins such as collagen, fibrinogen, fibronectin and vitronectin. These ECM proteins contain RGD sequences (Arg-Gly-Asp) that are recognised by the integrins on the cell surface. The attachment of these RGD peptides to the integrin initiates cell attachment and proliferation [74]. Thus, the biomaterial surfaces immobilised with these biologically active molecules have improved biocompatibility. 

### 4.1. Electrospinning

PHA nanofibres can be produced through electrospinning. It is important that the nanofabrication technique employed to fabricate nanofibres closely mimic the nanoscale topography of native ECM, thereby enhancing the interaction between cells and nanofibres to promote cell adhesion and proliferation [83]. Electrospinning is a popular approach in the fabrication of nanofibers. Electrospinning was first observed over a century ago by Rayleigh in 1897 and patented by Formals in 1934 [84]. In 1902, Gooley and Morton first designed the complete electrospinning unit [85]. 

Electrospinning is a cost-effective and versatile method to fabricate ultrafine fibres with special orientation [86,87,88]. The fibrous electrospun scaffolds offer significant advantages over other candidate materials, mainly because of their high surface-to-volume ratios, high porosity, and spatial interconnectivity, which are beneficial for cell migration and proliferation [85,89]. In this regard, electrospinning has gained attention as a potential technique to produce desirable scaffolds for biomedical applications as the nanofibres formed mimic the ECM [88]. Various parameters can affect the fabrication of the electrospun nanofibres including polymer concentration, volumetric flow rate, voltage, the solvent used and distance of the collector. The polymer concentration is a crucial parameter in electrospinning, as it defines the spinnability of a solution, thereby determining whether a fibre can be formed [88]. When the flow rate increases, the available high polymer volume increases the nanofibre diameter along with an increase in pore size.

The nanofiber construct of PHA-based scaffolds seems to be an attractive method in increasing and enhancing PHA applications in tissue engineering [59,90]. PHAs for nanoarchitecture has become a newly emerging trend for various applications especially biomedical applications [67]. The intrinsic nature of PHAs has enabled them towards fabrication of nanoparticles and nanocomposites. PHA nanofibers and nanoparticles have been extensively applied in various areas such as wound management [91], skin regeneration [92,93,94], drug delivery [95], antibacterial agents, nerve regeneration, orthopedic applications [96] and bioengineering [97]. It is believed that further chemical or biological modification of the fabricated nanofibrous scaffolds would improve the characteristics, especially biocompatibility to make this nanofibrous scaffold suitable in the biomedical field [98,99].

### 4.2. Aminolysis

Aminolysis is one of the widely used chemical modifications for surface biofunctionalisation, which introduces amine groups (R–NH_2_) and thiol groups (R–SH) as functional groups onto the target surfaces. Aminolysis involves the cleavage of the ester bonds to form amine groups and amine bonds on the surface [100]. Amine functionalities are generated by aminolysis on the PHA surfaces to conjugate with biomolecules [101]. Furthermore, the solvent used, and the duration of the treatment required are taken into consideration during aminolysis and depend on the type of PHA.

Numerous studies have been conducted to graft the surfaces of PHAs with a series of functional groups via aminolysis. Vigneswari et al. [102] showed that the immobilisation of fish-scale collagen peptides (FSCPs) onto the P(3HB-*co*-4HB) scaffold via aminolysis promoted the growth and adhesion of L929 mouse fibroblast cells.

Aminolysis is also suitable for the modification of unsaturated PHAs to transform PHAs into high value-added materials. Ma et al. [103], aminolysed poly(*N*-isopropylacrylamide) (PNIPAm) with a trithiocarbonate-based chain transfer agent (CTA) (PNIPAm-CTA) and *n*-butylamine to achieve a pendant thiol group at the end of the chain (PNIPAm-SH). Poly(3-hydroxydodecanoate-*co*-3-hydroxy-10-undecylenate) [P(3HDD-*co*-3H10U)] is an unsaturated PHA that has carbon-carbon double bonds which chemically react with PNIPAm-SH. The PNIPAm-SH tethered to P(3HDD-*co*-3H10U) film improved NIH 3T3-E1 mouse embryo fibroblast cell growth and proliferation. Furthermore, Yao et al. [104] performed the aminolysis of poly(2-dimethylamino-ethylmethacrylate) (PDMAEMA) to introduce thiol groups at the end of the chain, then grafted onto poly(3-hydroxydodecanoate-*co*-3-hydroxy-9-decenoate [P(3HDD-*co*-3H9D)] to form P(3HDD-*co*-3H9D)-*g*-PDMAEMA. P(3HDD-*co*-3H9D) is also an unsaturated PHA with double bonds on the polymer sidechains which allow further chemical modifications. As a result, P(3HDD-*co*-3H9D)-*g*-PDMAEMA exhibited enhanced proliferation of mouse embryonic fibroblasts (NIH 3T3).

Aminolysis has been proven as an efficient surface chemical modification for PHAs, improving the surface properties for better biocompatibility. It also provides a new way to develop functional PHA biomaterial for possible biomedical and biomedicine applications.

### 4.3. Biomolecule Immobilisation

Biomolecules including proteins such as collagen, chitosan, fibronectin, and peptides such as RGD peptides are biologically significant ligands. Numerous studies demonstrated that biomacromolecules, such as collagen, gelatin and chitosan, were immobilised on the surface of the polymer to enhance their properties. Moreover, RGD peptides composed of three amino acids, namely Arg-Gly-Asp, were also used as a short peptide sequence immobilised on the scaffold to determine distinct cellular adhesive properties and signal the binding sites to the integrins [105]. RGD peptides are the components of ECM proteins, including fibrinogen, collagen, vitronectin and fibronectin, involved in cell adhesion and specific binding to the transmembrane proteins [106,107]. RGD peptides are easily characterised with greater stability which makes them available to be potentially employed for biomaterial surface modification [106].

The reactive sites of the biomolecules should be compatible with the specific functionality of the scaffold surface to enable them to be covalently immobilised onto the surface [108]. The immobilisation of appropriate biomolecules onto the PHA scaffold surfaces can improve the scaffold properties, thereby forming good cell–scaffold interactions and govern cellular functions [109]. The presence of suitable biological molecules at the damaged site is important for effective tissue regeneration, as they effectively direct and guide cell adhesion, proliferation and differentiation via the regulation of metabolic pathways and gene expression [101]. Numerous studies have demonstrated that the incorporation of biomolecules with polymer mats provided good contact guidance to direct cell responses and behaviours for better tissue regeneration in biomedical applications. Table 3 shows the biomolecules immobilised onto different polymers for various biomedical applications.

## 5. Biomedical Applications of P(3HB*-co-*4HB)

A biomaterial is a benign material that can be adapted and applied in the biomedical fields [113]. According to European Society for Biomaterials (ESB), biomaterial is a material intended to interact with biological systems to augment, treat, evaluate or substitute any tissue, organ or function of the body [72]. Interestingly, P(3HB-*co*-4HB) has been widely used as a biomaterial due to its desirable properties. It can be tailored to address specific needs with great potential in tissue engineering. Furthermore, the properties of P(3HB-*co*-4HB) can be improved via surface modification or incorporation with appropriate biomolecules to increase its functions and effectiveness in biomedical application. Furthermore, natural polymers for scaffolds are inspired by the ECM that holds the cells together in a native tissue. Therefore, incorporating some materials, such as collagen (mainly type I and III that are found in the heart) and fibrin onto the scaffold surface has been investigated as an efficient approach in introducing functional groups on the scaffold surface. These functional groups are further bioconjugated with the biomolecules to enhance the cellular activities on the scaffolds [101].

**Figure 8:**
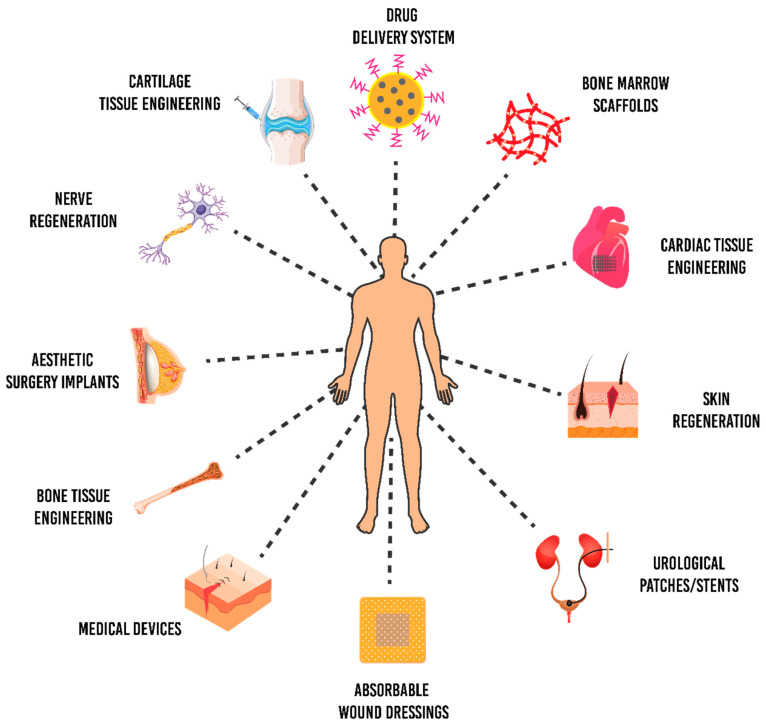
Schematic diagram of surface modified P(3HB-*co*-4HB) biomaterial and the applicability of the biopolymer in various biomedical applications. Tailoring the P(3HB-*co*-4HB) medical matrices similar to the biological functions helps to improve the tissue regeneration.

Tissue engineering exploits the concepts of life sciences and engineering to synthesise biological replacements used to repair, retain, replace and enhance tissue functions [114]. Generally, tissue engineering approaches have been applied in different tissues or organs such as bone, heart valve, blood vessels, liver, nerve and skin [84,85]. Most tissues or organs are deposited on the natural fibrous structures; thus tissue engineering applies this principle to fabricate promising biological substitutes which mimic the native ECM [88,115]. Hence, designing the P(3HB-*co*-4HB) medical matrices or substitutes similar to the biological functions helps to improve the tissue repair. Figure 8 shows the applicability of P(3HB-*co*-4HB) biomaterial in biomedical fields.

### 5.1. Cardiac Patch

The heart is a vital organ for human survival. The ECM of the heart plays vital roles in governing cellular functions including growth, survival, metabolism and differentiation for cardiac development [116].

The cardiac ECM is a complex network of fibres comprised of matrix proteins in which cardiac fibroblasts, myocytes, leukocytes and cardiac vascular cells reside [117]. Cardiovascular damage leads to the degradation of the cardiac ECM and the loss of functional myocardium, resulting in weakened contractility of the heart muscle that increases the risk of organ failure [118]. Hence, the introduction of functional cardiac tissue engineering is an advancement in the current therapy for the treatment of heart diseases.

Engineered cardiac substitutes have been designed to replace damaged tissue by providing a construct form of endogenous cardiac repair to promote the recovery of cardiovascular function. The development of a cardiac patch is a suitable alternative cardiac regenerative approach. In the current cardiovascular patch market, the available patches include GORE-TEX^®^ cardiovascular patch [119] and IMPRA^®^ ePTFE cardiovascular patch [120], which have been designed as heart tissue repair patches for implantation in the human heart. However, these cardiac patches are mostly composed of expanded polytetrafluoroethylene (ePTFE), a synthetic material that may more easily elicit thrombosis and negative immune responses [121]. Thus, the choice of biomaterial is still focused on the development of a good quality cardiac patch.

The desirable properties possessed by P(3HB-*co*-4HB) make it a good choice of biomaterial. Indeed, P(3HB-*co*-4HB) has been applied to fabricate engineered cardiac patches, blood vessel substitutes or vascular grafts. Qiu et al. [122] reported that the block poly(ester-urethane)s based on P(3HB-*co*-4HB) synthesised via melting polymerisation, exhibited non-cytotoxicity and promoted rat aortic smooth muscle cells (RaSMCs) cell growth and proliferation on the scaffold. Both platelet adhesion and plasma recalcification time evaluation demonstrated that the block poly(ester-urethane) based on P(3HB-*co*-4HB) possessed haemostasis ability which promoted rapid blood coagulation. Niu et al. [123] showed that mouse BMSCs were induced to differentiate into cardiomyocytes seeded on the P(3HB-*co*-4HB) patch, indicating that this scaffold supported the growth and attachment of differentiated cardiac cells.

### 5.2. Bone Grafts

A bone graft is a small piece of the bone substitute material transplanted into the skeleton to repair and rebuild the damaged bone [115]. Biodegradable P(3HB-*co*-4HB) has been developed into useful bone graft substitutes or scaffolds for filling the void space caused by the bone defects, as well as providing an anchorage for host cells to deposit bone repairs [87,124]. Bone tissue engineering which utilises an integration of a suitably designed scaffold is proven to regenerate new bone successfully [26,124]. The large surface-area-to-volume ratio and ultrathin fibre diameter of electrospun nanofibrous scaffolds have made them popular biomaterials applied widely in bone tissue engineering [87]. As an example, Fu et al. [125] showed that a P(3HB-*co*-4HB) electrospun fibre scaffold with a three-dimensional porous network enhanced the attachment and proliferation of BMSCs, improving the calvarial defects in vivo as the bone-like tissues and regenerated bone replaced the gradually degrading scaffold after eight weeks of implantation.

### 5.3. Nerve Regeneration

The nervous system comprises the central nervous system (CNS) and peripheral nervous system (PNS). The CNS consists of the brain and spinal cord, whereas the PNS consists of the ganglia and nervous tissue outside the CNS [18]. Axonal damage or nerve defects can cause severe body functional deficits because the nervous system functions to coordinate and transmit signals between different body parts [84,126]. P(3HB-*co*-4HB) biomaterial serves as a substrate for nerve tissue engineering. Designing the scaffold to possess appropriate chemical and mechanical properties is vital for the success of nerve tissue engineering for nerve tissue repairs or nerve regeneration [84]. The scaffold serves as structural support for the growth of nerve cells during nerve regeneration [18]. Xu et al. [127] fabricated P(3HB-*co*-4HB) into 3D nanofibre constructs via a novel phase separation process to mimic the natural ECM. The study demonstrated that a P(3HB-*co*-4HB) electrospun scaffold supported neural stem cells’ (NSCs) growth, adhesion and differentiation on the matrices, and hence is a promising candidate for the treatment of CNS injury.

### 5.4. Skin Grafts

The applicability of PHAs in the regeneration of skin after injuries or any damages is a novel approach for the treatment of skin defects. Various factors influence the materials and techniques used to reconstruct the skin integrity, including the severity and depth level of the injury, the sites of wound, the phase of the wound-healing process, the degree of microbial invasion, the type of drug taken by the patients and the health conditions of the patients [128,129]. P(3HB-*co*-4HB) deemed suitable to be developed as a biomaterial for designing wound dressing. This is based on a research by Shishatskaya et al. [128], whereby the mesenchymal stem cells (MSCs) exhibited better cell growth and proliferation on the non-woven membranes of degradable P(3HB-*co*-4HB). In vivo study showed that the non-woven membranes developed from P(3HB-*co*-4HB) reduced inflammation, improved the angiogenic properties of the skin and facilitated its healing process. Apart from that, Vigneswari et al. [59] revealed that electrospun nanofibres of a P(3HB-*co*-4HB)/collagen peptides matrix induced proliferation of mouse fibroblast cells (L929) on the construct and exhibited an accelerated wound closure in vivo which reached the highest percentage of 98% in 14 days. This proves that the nanofibrous P(3HB-*co*-4HB)/collagen construct can accelerate wound healing thus promising to be a desirable wound dressing.

### 5.5. Antimicrobial Scaffolds

The innovative concept of biomaterial scaffolds combined with antimicrobial activity plays a crucial role in supporting cell growth and proliferation, a major issue in healthcare. The tissue-engineered scaffolds that mimic the chemical composition and physical structure of native ECM helps damaged cells and tissue to recover by providing coverings and helping dermal tissue regeneration. Additionally, these scaffolds have a high surface-to-volume ratio and high porosity. P(3HB-*co*-4HB) copolymer as a scaffold biomaterial elucidates appropriate host tissue responses. The novel P(3HB-*co*-4HB) nanofibre scaffold fabricated via electrospinning and surface functionalised with RGD peptide enhances H9c2 myoblast cell attachment and proliferation, playing a major role in the repair and regeneration of infracted cardiac tissues [99]. These scaffolds have been widely applied in bone tissue engineering, the composite P(3HB-*co*-4HB)/GO (graphene oxide) scaffold promotes regeneration of bone in rats [130]. These scaffolds were improved with cellular performance and biodegradable. Similarly, P(3HB-*co*-4HB) supports osteoblast differentiation of human MSC compared to poly (3-hydroxybutyrate-*co*-3-hydroxyhexanoate) and polylactic acid. The octacalcium phosphate incorporated P(3HB-*co*-4HB) nanofibrous scaffolds fabricated by electrospinning facilitate the osteogenic differentiation of MSCs and bone regeneration [131]. For effective regeneration of injured skin, novel material scaffolds are required to fulfil the antimicrobial potential, have no toxic effects, provide good mechanical support and sufficient elasticity to fit the wound shape. Shishatskaya et al. [132] reported that P(3HB-*co*-4HB) intended for skin repair does not change the biological value of human blood cells. Also, P(3HB-*co*-4HB) has no toxic effect on the membrane and blood cells, therefore the development of P(3HB-*co*-4HB) scaffolds has the potential to meet the requirements for tissue regeneration and bioengineering.

### 5.6. Drug-Delivery System

The drug-delivery system is designed for the bioactive compound for the optimal bioavailability; therefore, they provide a prolonged time of action and therapeutic concentrations. The formation of such a drug-delivery system relies on the polymer depending on physiochemical, mechanical properties, polymer-based formulation process that includes drug solubilisation and drug protection from the deactivation and degradation as the result of drug interaction with biomolecules [133,134]. These drug-delivery systems are designed as the drug delivery carriers for the transportation of drug to the site of action and controlled drug release.

PHAs play a smart role in drug-delivery applications since they hold their proven biocompatibility, biodegradability and non-toxicity properties. According to the study by Fisol et al. [135] it is possible to load nanoparticles using the biodegradable polyester P(3HB-*co*-4HB). The anticancer drug Docetaxel was successfully encapsulated into nanoparticles without affecting the physiochemical properties. The release of such a profile is influenced by drug/polymer ratio, where P(3HB-*co*-70%4HB) polymer were loaded inside the nanoparticle with no aggregation. The aforementioned properties show P(3HB*-co-*4HB) can be a drug carrier candidate for controlled release application in medicine. Previous study also investigated nanoparticles from P (3HB*-co-*4HB) copolymer encapsulated with Rifampicin, an antituberculosis drug. In addition to this type of nanoparticle, a type of coated nanoparticles was also produced by using chitosan as the coating material. Therefore, P(3HB-*co*-4HB) has potential for further development as a drug-delivery system in the future.

## 6. Challenges and Outlook

The advantages of PHA and their applications as biomaterials have brought forth many successful inventions and innovations in the biomedical field. Nevertheless, there some underlying challenges for mass production and consumption of PHA which requires relevant considerations. One of the major limitations high cost of PHA production through bacterial fermentation. Researchers are still unravelling to design optimal upstream and downstream processes for efficient and cost effective PHA production. The main cost absorbing factor being carbon feedstock and the PHA extraction method.

The nanofabrication of PHA through electrospinning is a challenge especially when it involves fabrication of uniform nanofibers repeatedly without altering its superior properties. In order to improve the morphology and properties of nanofiber formation, electrospinning innovations in diversifying the setup such as coaxial electrospinning, side-by-side electrospinning and emulsion electrospinning have been carried out.

The development of electrospun nanofibers in the drug-delivery system have been carried out with the synergic effects with the fabrication technology of a nanofiber matrix. The means of adjusting the fabrication patterns, matrix thickness, matrix composition and structural features could affect the overall rate of drug delivery to targeted sites. In vivo stability and efficient controlled drug delivery by nanofiber matrix should be further investigated. It is foreseen that further studies will include the application of a nanofiber matrix with different drug combinations and their efficient applicability on different parts of the living systems. Nanofibers can be designed to fit in small and hard to reach areas in living systems as they are flexible and possess high surface area to volume ratio.

A PHA matrix is capable of promoting the growth, adhesion, proliferation and differentiation of mesenchymal stem cells (MSCs). The promising features of MSCs include their regenerative properties and ability to differentiate into diverse cell lineages and this has gained great interest on the stem cell-based scaffold for treatment of diseases. There is a vast of potential investigations of the differentiated MSCs on various modified PHA matrix to be conducted. Further in vitro study as well in vivo study on the cell–scaffold interactions, tissue repair metabolism, inflammatory responses and scaffold degradation evaluation can be investigated.

The ever versatile PHA in its many different forms has been investigated for a wide range of application, especially for medical purposes. The advances in physical, chemical and biological processes to tailor the surface architecture of this biopolymer is transforming PHA’s compatibility to cater for a broad range of healthcare treatments that include heart disease treatment, cancer therapy, metabolic disorders, regenerative medicine, neurodegenerative and more medical therapies. There is much more to explore using this unique and durable biomaterial and the recent research is a testament to its functionality and future prospective.

## Figures and Tables

**Table 1:** The general properties of the common types of PHAs with a wide range of values [4,41,42,43,44].

Property	P(3HB)	P(3HB-*co*-4HB)
Glass transition temperature, T_g_	2–4 °C	−48–4 °C
Melting temperature, T_m_	160–175 °C	50–175 °C
Tensile strength, σ	15–40 MPa	17–104 MPa
Young’s modulus	1–2 GPa	0.07–1.5 GPa
Elongation at break	1–15%	14–1320%
Crystallinity	50–80%	34–60%
Common physical form	Film, microfiber, microparticle	Film, microfibre, microparticle

**Table 2:** The different types of P(3HB-*co*-4HB) producing bacteria.

Bacteria	PHA Composition	Authors and Numbers in Reference List	Year
*Comamonas acidovorans*	P(3HB-*co*-4HB)	Lee, Azizan and Sudesh [52]	2004
*Cupriavidus necator*	P(3HB-*co*-4HB)	Rao, Kumar, Balaji, and Sehgal [53]	2010
Transformant *Cupriavidus* sp. USMAA1020	P(3HB-*co*-4HB)	Norhafini, Thinagaran, Shantini, Huong, Syafiq, Bhubalan and Amirul [54]	2017
*Ralstonia eutropha* strain A-04	P(3HB-*co*-4HB)	Chanprateep, Katakura, Visetkoop, Shimizu, Kulpreecha and Shioya [55]	2008
*Wautersia eutropha* H16	P(3HB-*co*-4HB)	Kimura, Ohura, Matsumoto and Ikarashi [56]	2008
*Cupriavidus* sp. TMT 11	P(3HB-*co*-4HB)	Chai, Sadasivam and Vigneswari [57]	2019

**Table 3:** List of biomolecules immobilised onto P(3HB-*co*-4HB) to enhance the properties of the material for biomedical applications.

Biomolecules on P(3HB-*co*-4HB)	Potential Applications	Enhanced Properties	References
Collagen	Fibroblast cells (L929) adhesion and proliferation	Hydrophilicity of P(3HB-*co*-4HB)/collagen blend scaffolds increased as the collagen concentration increased and the cell adhesion and growth of L929 culture enhanced.	[43,77]
Gelatin	Fibroblast cells (L929), mesenchymal stromal culture adhesion and proliferation	Addition of gelatin improved the surface wettability of the scaffolds as well as biological properties; cell adhesion, proliferation, differentiation	[98,110]
Zein	NIH3T3 fibroblast cells and MG-63 osteoblast cells adhesion and proliferation	Improvement in tensile strength and increase in elongation at break as well as improved cytocompatibility	[94]
Bacterial cellulose	Chinese Hamster Lung (CHL) fibroblast cells attachment and proliferation	The biocompatibility and cell adhesion of CHL cells of the composite scaffold was enhanced	[111]
Chitosan	Wound healing	The hydrophobic nature of the P(3HB-*co*-4HB) copolymer was modified with chitosan. Water-absorption capacity and solubility increased	[112]

## Data Availability

The data presented in this study is openly available.
